# Associations Between Neighborhood SES Disadvantage and Feelings of Depression and Loneliness in Older Adults

**DOI:** 10.1093/geroni/igab046.3296

**Published:** 2021-12-17

**Authors:** Dextiny McCain, Adrienne Aiken Morgan, Regina Wright

**Affiliations:** 1 North Carolina Central University, Durham, North Carolina, United States; 2 North Carolina A&T State University, Greensboro, North Carolina, United States; 3 University of Delaware, University of Deleware, Delaware, United States

## Abstract

Previous research suggests depressive symptoms and loneliness are increasingly prevalent among older adults living in lower-income neighborhoods. The purpose of this study was to examine the extent to which neighborhood socioeconomic status (SES) was associated with depressive symptoms and loneliness among a sample of older adults from the Healthy Heart and Mind Study (N = 165; mean age = 68.48 (SD = 6.26); 66.7% women; 40.6% African American). It was hypothesized that older adults living in neighborhoods with greater socioeconomic disadvantage would report more depressive symptoms and loneliness than those residing in neighborhoods with less socioeconomic disadvantage. Depression was assessed with the Beck Depression Inventory-II (BDI-II), and loneliness was assessed using the Revised University of California, Los Angeles (UCLA) Loneliness scale. Neighborhood SES was measured with the Area Deprivation Index (ADI), which allows rankings of neighborhoods by SES disadvantage both statewide and nationally. After controlling for demographic variables (age, sex, and race), linear regression analyses showed that greater neighborhood SES disadvantage was associated with higher depression scores (β = -.094; p = .041) and higher loneliness scores (β = -.258; p = .003). These findings highlight the importance of neighborhood context on mental health in older adults, as underserved populations are more likely to experience declines in mental health under strenuous circumstances. Future research should investigate the impact of neighborhood SES on mental health in aging adults.

